# 

**Published:** 1898-08

**Authors:** 


### CONTENTS.

ORIGINAL COMMUNICATIONS—

Some Recent Surgical Work. By Hunter P. Cooper, M.D., Atlanta, Ga........... 361

The Conditions of Imperfect Development of the Septum between the Mouth and Naso-Pharynx, Usually Termed Cleft Palate. By M. F. Carson, M.D., Griffin, Ga........ 367

Infant Feeding. By G. P. Robinson, M.D., Atlanta, Ga.......... 373

CORRESPONDENCE—

The Color of the Negro when Born.......... 384

EDITORIAL—

The New Cure for Consumption.......... 385

Physicians' Accounts.......... 387

Consolidation of Medical Colleges.......... 389

NEWS AND NOTES.......... 391

BOOK REVIEWS, PAMPHLETS, EXCHANGES—

Exchanges.......... 397

Book Reviews.......... 398

SELECTIONS AND ABSTRACTS.......... 402

MEDICAL ITEMS—Continued.......... x, xi, xii, xiii, xiv, xvi

ADVERTISERS' INDEX TO ADVERTISEMENTS.......... xviii

